# Hard-templating of chiral TiO_2_ nanofibres with electron transition-based optical activity

**DOI:** 10.1088/1468-6996/16/5/054206

**Published:** 2015-10-22

**Authors:** Cui Wang, Shaohua Liu, Yingying Duan, Zhehao Huang, Shunai Che

**Affiliations:** School of Chemistry and Chemical Engineering, State Key Laboratory Metal Matrix Composites, Shanghai Jiao Tong University, 800 Dongchuan Road, Shanghai, 200240 People’s Republic of China

**Keywords:** anatase, hard-templating method, electron transition-based optical activity, circular dichroism

## Abstract

The fabrication of optically active inorganic nanomaterials with chiral superstructures attracts attention because of their potential applications in chemical sensing and non-linear optics. Here, we present a facile way to prepare TiO_2_ nanofibres, in which the nanocrystals are helically arranged into a chiral superstructure. Notably, the chiral superstructure shows strong optical activity due to the difference of absorbing left- and right-handed circularly polarized light. This special optical activity resulted from electron transition from the valence band to the conduction band of TiO_2_ through a vicinal effect of helically arranged TiO_2_ nanocrystals.

## 1. Introduction

Optically active materials respond differently to left and right-handed circularly polarized light in terms of propagation speed and extinction. Optically active inorganic materials have aroused general interest from synthetic, structural, and functional standpoints because of their potential applications in chiral separations, chemical and biological sensors, and optical devices [1–11]. As is known, electronic transition-based absorptions in the ultraviolet (UV) or visible range are very common in compounds, and organic materials with electron transition-based optical activity (ETOA) have been investigated extensively. However, so far, inorganic materials with ETOA have been scarcely reported [12–15]. By comparison, chiral inorganic materials with ETOA have a clear advantage for application over both chiral organic assemblies and chiral nanoparticles supported by organics due to their increased physical robustness, resulting in permanent molecule or block chirality.

On the other hand, self-organization of entities in multidimensional scales can lead to unexpected emergence of collective intrinsic properties, which are neither those of the corresponding bulk phase nor those of the isolated particles. Collective optical and magnetic properties have been observed for the dipolar interactions between inorganic nanocrystals when they are organized into 2D superlattices. To find new physical properties as a result of the ordering, it is prerequisite for the nanocrystals to be ordered at the mesoscopic scale [16]. Furthermore, the monodispersed blocks of colloid nanocrystals of silver, CdSe, cobalt, and gold have been built into ordered superstructures by surfactant-templated approaches [17–19]. However, soft-template methods often suffer from the elaborate choice of template, solvent and precursor, because precursors of many materials either condense too fast, destroy the assembly of the surfactant, or are not compatible with aqueous synthesis. For the preparation of ordered nanoarchitecture, hard templating has some advantages compared with soft templating, especially in its stability, predictability, controllability and versatility [20–22].

Herein, we present a simple hard-templating method for constructing porous helical nanofibre superstructures using inorganic anisotropic nanocrystals, which features the collective intrinsic property of ETOA. This method involves directly casting metal oxides on the helical carbonaceous nanotubes, and subsequently removing the template along with the formation of metal oxide nanocrystals by calcination at high temperature in air. The resulting materials with helical stacking of nanocrystals have distinct and durable ETOA, responding to circularly polarized light. Moreover, such hierarchical materials possess high special surface area and large pore volume, and pores come from the removal of the hard template in the axial direction as well as the looser packing of nanocrystals in the wall, respectively.

In this investigation, TiO_2_ was selected as an example for illustrating our proposal, because the anatase nanocrystals typically show ultraviolet absorption in the region of 200–400 nm, which would exhibit ETOA if they had an ordered spatial arrangement with suitable pitch [12, 23]. Here, the synthesis process of chiral TiO_2_ nanocrystals is as follows: firstly, chiral polypyrrole nanotube (CPPyN) and chiral carbon nanotube (CCN) were synthesized by the method of our previous report [24–26]. Both of the core/shell coaxial chiral nanotubes of CPPyN/TiO_2_ and CCN/TiO_2_ were obtained by nanocasting with a thin TiO_2_ layer. Upon removal of the CPPyN or CCN templates, TiO_2_ nanotubes were obtained as free-standing stacks of anatase nanocrystals that have a long-range chiral helical structure, resulting in ETOA properties.

## 2. Experimental details

### 2.1. Chemicals

Titanium(IV) butoxide (TBOT, 99% solution), pyrrole, and ammonium persulfate (APS) were purchased from Sinopharm Chemical Reagent Co. Ltd, China. N-stearoyl-L/D-glutamic acid (C_18_-L/D-Glu) was purchased from Ajinomoto Co. Ltd.

### 2.2. Preparation of chiral templates of CPPyN and CCN

Chiral polypyrrole nanotubes were synthesized by self-assembly of C_18_-L/D-Glu and pyrrole. Typically, the synthesis molar composition was: C_18_-l-Glu: pyrrole: methanol: H_2_O: APS = 1:40:5351:57081:40. 0.06 mmol of C_18_-l-Glu was dissolved in 10.2 g methanol at room temperature, then 2.4 mmol pyrrole and 60 mL deionized water were added. The solution was stirred for 10 min, then a precooled aqueous solution of APS (2.4 mmol in 1.2 mL deionized water) was added to the mixture and stirred for a further 30 min. A brown solid CPPyN was obtained after filtrating and washing three times with H_2_O and C_2_H_5_OH, and dried for 12 h at 40 °C in a vacuum. For carbonization of the CPPyN, the above products were heated at a rate of 1.5 °C min^−1^ to 900 °C under flowing Ar, held for 6 h. After slowly cooling the sample to room temperature, chiral carbon nanotubes were obtained.

### 2.3. Preparation of chiral TiO_2_ nanotubes

20 mg chiral carbon nanotubes (CPPyNs) or chiral carbon nanotubes (CCNs) were added to 1 g TBOT, then stirred for 1 h at room temperature. The products were washed by 10 mL ethanol under N_2_ atmosphere, collected by centrifugal separation and dried for 12 h at 60 °C. Then the core/shell (CPPyN/TiO_2_ and CCN/TiO_2_) coaxial antipodal chiral nanotubes of L/R-TiO_2_@CPPyN and L/R-TiO_2_@CCN (L and R denote left- and right-handed) were heated at a rate of 1.5 °C min^−1^ to 550 °C under the air and kept for 6 h. Then, the chiral TiO_2_ nanofibres were obtained (the samples obtained by calcination of L/R-TiO_2_@CPPyN and L/R-TiO_2_@CCN were denoted as L/R-P-TiO_2_ and L/R-C-TiO_2_, respectively).

### 2.4. Characterization

The microscopic characteristics of the samples were observed via scanning electron microscopy (SEM, JEOL JSM-7800F Prime) at 1.0 kV. High-resolution transmission electron microscopy (HRTEM) was performed with a JEOL JEM-2100 microscope operated at 200 kV. Powder x-ray diffraction (XRD) patterns were recorded on a Rigaku x-ray diffractometer D/MAX-2200/PC using Cu K radiation (40 kV, 20 mA). The N_2_ adsorption/desorption isotherms were obtained at 77 K by using a Quantachrome NOVA 4200E surface area and pore-size analyser. Diffuse reflection circular dichroism (DRCD) and diffuse reflection ultraviolet–visible (DRUV–vis) spectra were taken using a JASCO J-815 spectropolarimeter fitted with DRCD apparatus.

## 3. Results and discussion

**Figure 1.**
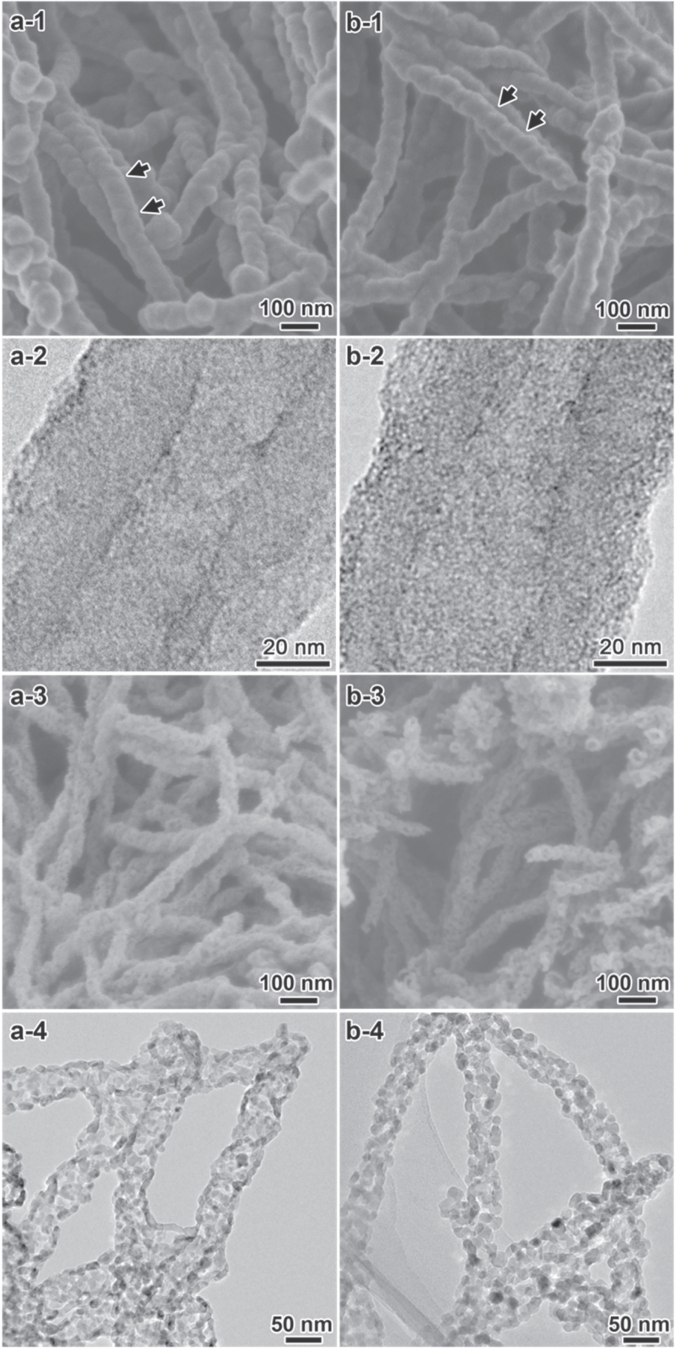
SEM and TEM images of the chiral L-TiO_2_@CPPyN (a-1) and (a-2), L-TiO_2_@CCN (b-1) and (b-2), L-P-TiO_2_ (a-3) and (a-4) and L-C-TiO_2_ (b-3) and (b-4).

**Figure 2.**
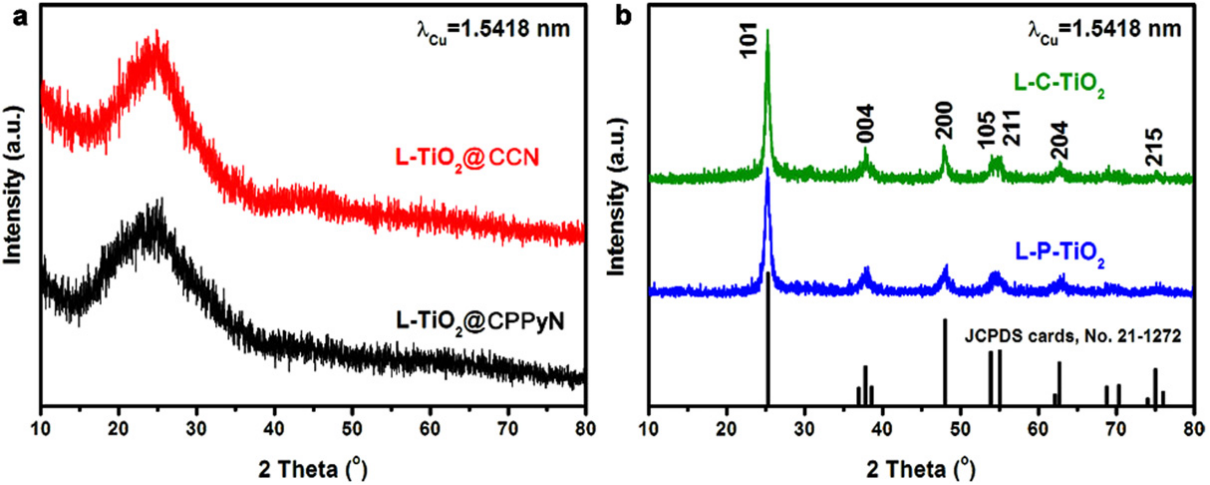
XRD patterns of the samples shown in figure 1.

Both of the core/shell coaxial chiral nanotubes of antipodal L/R-TiO_2_@CPPyN and L/R-TiO_2_@CCN were obtained by nanocasting of a thin TiO_2_ layer on the chiral nanotubes of CPPyN and CCN by controlled hydrolysis and condensation of TBOT with H_2_O under conditions of ambient pressure and temperature, where the helical nanotubes of CPPyN or CCN can serve as hard templates. Figure 1 shows SEM and TEM images of L-TiO_2_@CPPyN, L-TiO_2_@CCN, L-P-TiO_2_ and L-C-TiO_2_ derived from L-CPPyN and L-CCN (figure S1). Here, the L-CPPyN with ∼90 nm in diameter and L-CCN with ∼85 nm in diameter were used as templates. With uniform encapsulation of the TiO_2_ nanoshell around L-CPPyNs or L-CCNs, the double-helical morphology of the templates was well maintained (figure 1). The as-prepared L-TiO_2_@CPPyN or L-TiO_2_@CCN hybrids synthesized with left-handed CPPyNs or CCNs were exclusively composed of left-handed double-helical fibres. The right-handed R-TiO_2_@CPPyN hybrid nanotubes gave mirror image profiles with the same size and quality (figure S2). The diameters of the fibres increased from ∼90 nm of L-CPPyNs to ∼100 nm in L-TiO_2_@CPPyNs, and from ∼85 nm of L-CCNs to ∼95 nm in L-TiO_2_@CCNs (figure S3), indicating that such mineralization yielded highly full coverage of about ∼5 nm average thickness of TiO_2_ nanoshell over the entire length of the templates. In addition, the fibres possess inner tubes with a diameter of ∼20 nm that are constant along the central axis of each individual tube (figures 1(a-2), (b-2)), which implied the presence of the helical lipid aggregation of *N*-acyl-l-glutamic acid (C_18_-l-Glu) in the CPPyNs or CCNs. From the distinct helical morphology, the pitch length along the axis of the fibre was estimated to be ∼100 nm (shown as arrows in figure 1). TiO_2_ and carbon could not be distinguished from TEM images (figures 1(a-2), (b-2)), because TiO_2_ attached uniformly on the surface of CPPyNs and CCNs is in the form of an amorphous structure, which is the same as helically arranged carbonaceous nanostructured building blocks of CPPyNs and CCNs consisting of graphitic and amorphous carbon [25].

**Figure 3.**
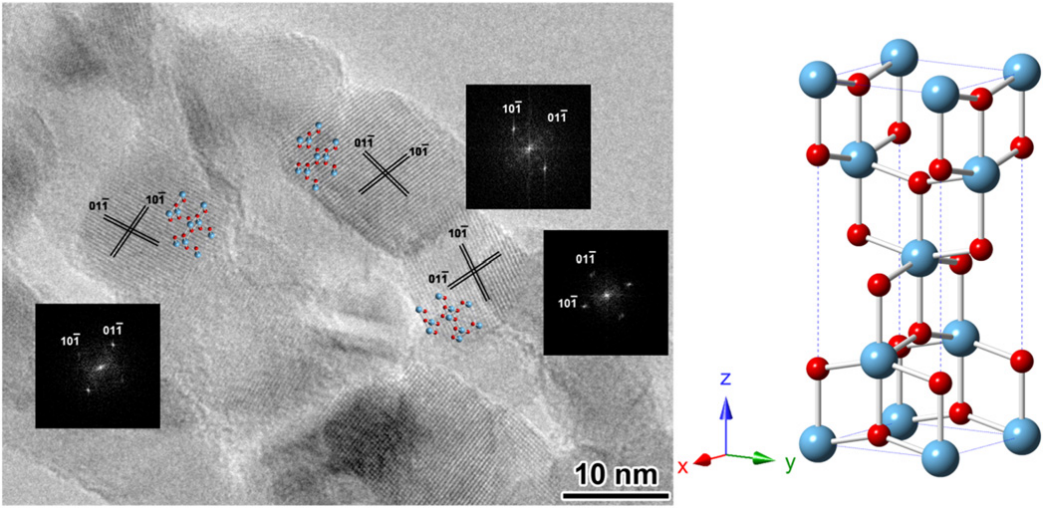
The HRTEM image and the corresponding Fourier diffractograms (FDs) of three nanocrystals in the L-P-TiO_2_ nanofibres shown in figure 1(a-3).

As shown in figures 1(a-3) and (b-3), upon removal of CPPyNs or CCNs, TiO_2_ nanofibres with the helical morphology were obtained, which indicated those free-standing TiO_2_ nanofibres have long-range chiral helical structure with the arrangement of nanoparticles. The diameters of fibres decreased from ∼100 nm of L-TiO_2_@CPPyNs to ∼60 nm in L-P-TiO_2_, and from ∼95 nm of L-TiO_2_@CCNs to ∼55 nm in L-C-TiO_2_. However, the rotational misplaced arrangement of TiO_2_ nanocrystals still remained and further enhanced circular dichroism.

Figure 2 shows XRD patterns of L-TiO_2_@CPPyN, L-TiO_2_@CCN, L-P-TiO_2_ and L-TiO_2_@CCN. As shown in figure 2(a), the XRD patterns of the as-prepared L-TiO_2_@CPPyN and L -TiO_2_@CCN show only a broad band at 2*θ* = 25°, indicating that the TiO_2_ coated on CPPyN or CCN was amorphous. However, after calcination at 550 °C in the air, the XRD patterns exhibited identical reflections indexed as the anatase phase of TiO_2_ [JCPDS cards, No. 21-1272] with the space group of *I*4_1_/*amd* and lattice parameters of *a* = 3.79 Å and *c* = 9.51 Å, [23, 27, 28] and no other peaks related to impurities, such as rutile or brookite were detected. The mean crystallite sizes of L-P-TiO_2_ and L-C-TiO_2_ were under ∼10 nm, which were evaluated using Debye–Scherrer’s formula [29]. By comparison, the size of the anatase nanocrystals formed by the hard templating route here is smaller than that of the chiral TiO_2_ nanofibres synthesised by the surfactant-templating method, which could be attributed to their confined growth on the helical surface of templates as well as a small amount of coating of TBOT as the titanium precursor [12].

The HRTEM image in figure 3 shows that the TiO_2_ nanotubes were composed of loosely packed anatase nanocrystals. The diameters of the nanocrystals are less than 10 nm, which agreed with the XRD results. The possible reason for these anatase 2D nanosheets is that the interface TiO_2_ coating close to the carbonaceous tubes has to grow restrictively and crystallize along the template surface, and different conditions with the outside TiO_2_ cause their anisotropic growth into nanosheet structures. These nanocrystals exhibit the contrast close to 〈111〉 zone axis of the anatase structure, which indicates that the nanocrystals must have a preferential growth direction during the crystallization process. Additionally, it has been observed that such anatase 2D nanosheet structures are not favoured to be directly formed according to the Wulff construction, because these anatase crystals will be prone to grow into the octahedral shape with the majority of the exposed surface of (101) facets for the energetic stability [30–35]. Nevertheless, the adjacent nanocrystals can grow and crystallize with a rotational arrangement according to the demand of the helical template surface, even if the hard templates have been removed in the stage with elevated temperature in air.

**Figure 4.**
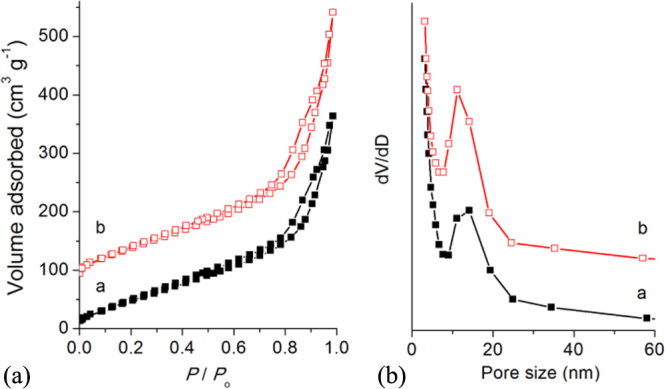
Nitrogen adsorption–desorption isotherms and pore size distributions of L-P-TiO_2_ (a) and L-C- TiO_2_ (b). The isotherm b is offset vertically by 80 cm^3^ g^−1^ STP.

High-surface-area TiO_2_ nanocrystals are of particular interest for applications such as dye-sensitized solar cells, photocatalysts, gas sensors, and batteries [36–39]. Because of the extraordinarily large surface areas and abundant channels or pores, the nanoporous materials can greatly facilitate mass diffusion within frameworks. Nitrogen adsorption measurements were performed on the chiral TiO_2_ samples (figure 4). The Brunauer–Emmett–Teller surface areas of TiO_2_ nanofibres obtained from L-TiO_2_@CPPyN and L-TiO_2_@CCN are 194 and 224 m^2^ g^−1^, respectively, with corresponding pore volumes of 0.539 and 0.689 cm^3^ g^−1^ and peak pore diameters of 15 nm. According to the results by the above SEM and TEM characterization, their pores would be created from the removal of the hard template in the axial direction as well as the looser packing of nanocrystals in the hierarchical wall, respectively. The specific surface area and pore volumes of TiO_2_ templated by chiral carbonaceous nanotubes compare well with TiO_2_ that has been templated by other mesoporous hosts [40–42]. Therefore, the above results demonstrate that such a helical template can also be a new candidate for the synthesis of porous materials with higher specific surface area and larger pore volume, but not just limited to mesoporous templates.

**Figure 5.**
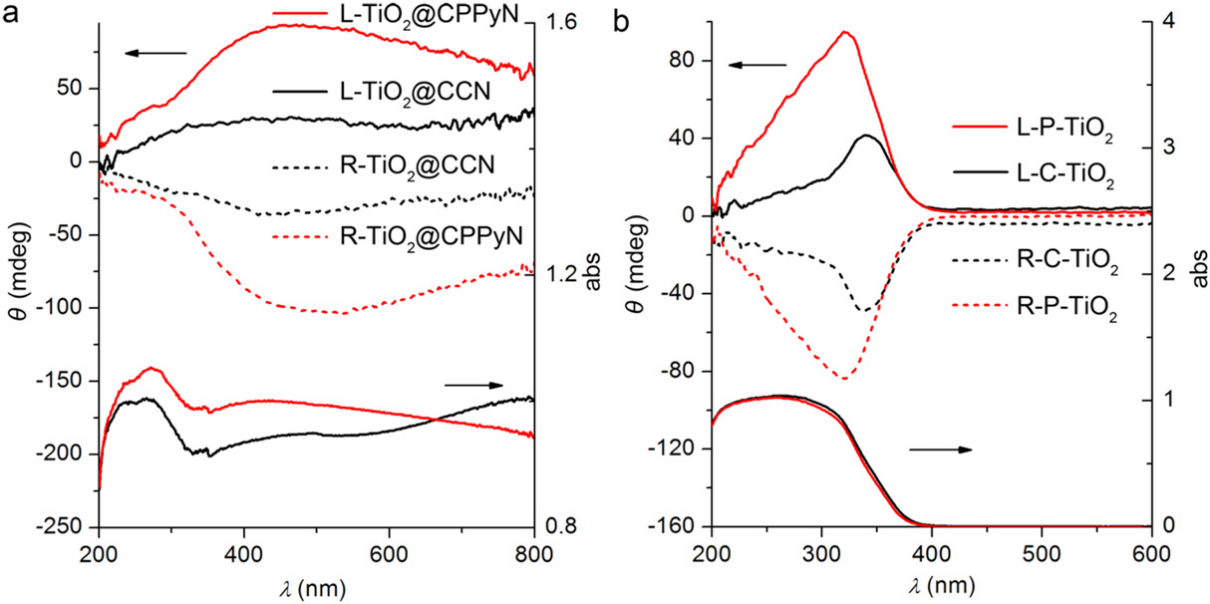
UV–vis and DRCD spectra of antipodal as-prepared hybrid materials templated by PPy (L/R-TiO_2_@CPPyN) and carbon nanotubes (L/R-TiO_2_@CCN) and calcined (L/R-P-TiO_2_ and L/R-C-TiO_2_) chiral TiO_2_ nanotubes.

As is known, it still remains a fundamental challenge to organize the anisotropic nanocrystals into ordered structures with collective properties, such as chemical, physical (optical, electrical, magnetic, etc) properties. The optical activity of materials shows their ability to have differential interactions with left- and right-handed circularly polarized light, resulting from their interaction between their chiral structure and light passing through, which can be detected by solid-state DRCD. Figure 5(a) shows the DRCD and UV–vis spectra of two pairs of antipodal L/R-TiO_2_@CPPyN and L/R-TiO_2_@CCN. For the above hybrid materials, the DRCD and UV–vis spectra show the composite signals, which are attributed to TiO_2_ and carbonaceous materials of L/R-CPPyN or L/R-CCN. The signals in the range of less than 350 nm are mostly attributed to the outside TiO_2_ on the template, while a broad absorption band in the range of 200–800 nm is attributed to CPPyN or CCN resulting from the combination of nanoeffect, polarization, and π–π conjugated structure, as described in our previous work [25]. After the removal of the carbonaceous template, the antipodal L/R-P-TiO_2_ and L/R-C-TiO_2_ showed strong mirror-image CD signals with a peak at 330–350 nm corresponding to the UV absorption edges of TiO_2_ (figure 5(b)). This ETOA can be considered a result of the dynamic coulomb interaction under the dissymmetric field electron transition from the valence band to the conduction band of TiO_2_ through a vicinal effect of helically arranged TiO_2_ species, resulting in their selective reflection of left- or right-handed circularly polarized light in the ultraviolet absorption region [12–14]. In addition, the CD signal of L/R-TiO_2_@CPPyN is stronger than L/R-TiO_2_@CCN. The DRCD intensity of L/R-P-TiO_2_ is much stronger than that of L/R-C-TiO_2_. One possible reason is the poor helical morphology of CCNs, the other might be the stronger interaction between Ti(IV) and amino groups of CPPyNs than physical attachment with CCNs, resulting in more closely stacked TiO_2_ nanocrystals in CPPyN and further inducing stronger dynamic coulomb interaction and CD signals [10, 43]. A recent report indicates that the chiral nematic organization of TiO_2_ nanocrystals into a mesoporous film can also endow them with new a new ability to reflect circularly polarized light, however, which can be ascribed to the scattering at the inorganic/air interfaces of the helical arrays rather than the electron transition [5].

## 4. Conclusions

In summary, chiral TiO_2_ nanofibres with ETOA can be easily synthesized by chiral helical hard templates. Such materials with hierarchical structure possess high special surface area and large pore volume from the removal of the hard template in the axial direction as well as the looser packing of the nanocrystals in the wall, respectively. The materials selectively reflect left- and right-handed circularly polarized light, which indicates a chiral organization of the titania crystallites. These novel highly porous titania nanotubes may be excellent materials for applications in dye-sensitized solar cells, photocatalysts, and lithium ion batteries. Therefore, one can reasonably expect that other porous materials with ETOA could be prepared by this simple hard templating method.
